# Polygenic Risk for Schizophrenia Has Sex-Specific Effects on Brain Activity during Memory Processing in Healthy Individuals

**DOI:** 10.3390/genes13030412

**Published:** 2022-02-24

**Authors:** Elise Koch, Lars Nyberg, Anders Lundquist, Karolina Kauppi

**Affiliations:** 1 Department of Integrative Medical Biology, Umeå University, 901 87 Umeå, Sweden; lars.nyberg@umu.se (L.N.); karolina.kauppi@umu.se (K.K.); 2 Umeå Center for Functional Brain Imaging, Umeå University, 901 87 Umeå, Sweden; anders.lundquist@umu.se; 3 Department of Radiation Sciences, Diagnostic Radiology, University Hospital, Umeå University, 901 87 Umeå, Sweden; 4 Department of Statistics, School of Business, Economics and Statistics, Umeå University, 901 87 Umeå, Sweden; 5 Department of Medical Epidemiology and Biostatistics, Karolinska Institute, Nobels väg 12A, 171 65 Solna, Sweden

**Keywords:** memory processing, brain activity, fMRI, polygenic risk, schizophrenia, sex differences, dorsolateral prefrontal cortex, superior parietal lobule

## Abstract

Genetic risk for schizophrenia has a negative impact on memory and other cognitive abilities in unaffected individuals, and it was recently shown that this effect is specific to males. Using functional MRI, we investigated the effect of a polygenic risk score (PRS) for schizophrenia on brain activation during working memory and episodic memory in 351 unaffected participants (167 males and 184 females, 25–95 years), and specifically tested if any effect of PRS on brain activation is sex-specific. Schizophrenia PRS was significantly associated with decreased brain activation in the left dorsolateral prefrontal cortex (DLPFC) during working-memory manipulation and in the bilateral superior parietal lobule (SPL) during episodic-memory encoding and retrieval. A significant interaction effect between sex and PRS was seen in the bilateral SPL during episodic-memory encoding and retrieval, and sex-stratified analyses showed that the effect of PRS on SPL activation was male-specific. These results confirm previous findings of DLPFC inefficiency in schizophrenia, and highlight the SPL as another important genetic intermediate phenotype of the disease. The observed sex differences suggest that the previously shown male-specific effect of schizophrenia PRS on cognition translates into an additional corresponding effect on brain functioning.

## 1. Introduction

Schizophrenia is a severe neuropsychiatric disorder that affects about 1% of the population [1]. Cognitive impairment is a central feature of schizophrenia [2,3], and a wealth of research has highlighted impaired working memory (WM) [4,5,6] and episodic memory (EM) [3,7] as core domains of dysfunction in the disease. Genetics are known to play a crucial role in the development of schizophrenia, with an estimated heritability of approximately 80% [8,9]. The largest schizophrenia genome-wide association study (GWAS) performed by the Psychiatric Genomics Consortium (PGC) [10] has identified a large number of genetic risk variants that individually have weak effects on the phenotype [11]. A polygenic risk score (PRS) can be calculated to examine the impact of cumulative genetic risk for schizophrenia on related phenotypes [12,13]. The association between schizophrenia PRS and lower cognitive ability in unaffected individuals is well-documented [14]. However, recent evidence suggests that schizophrenia PRS is associated with lower cognitive ability in males only [15,16]. Sex differences are known to exist in schizophrenia patients, with males having an earlier age at onset, worse treatment response to antipsychotics [17,18,19,20], and being affected more frequently and more severely than females [21,22]. Moreover, it has been reported that male schizophrenia patients have worse negative and cognitive symptoms than female patients [23,24,25,26,27,28], which could be due to sexual dimorphisms in brain structure and volume [29,30] as well as sex hormone differences [30,31]. Thus, it is possible that schizophrenia genetics impact cognition through interactions with biological processes that differ between the sexes, but whether such sex*schizophrenia PRS interactions translate into an effect on related intermediate phenotypes has not yet been investigated. Functional brain activity constitutes an intermediate phenotype that allows disease genetics to be mapped onto related brain processes that are typically altered in schizophrenia patients [32]. Neuroimaging studies using functional magnetic resonance imaging (fMRI) have revealed dysfunctional activation of the dorsolateral prefrontal cortex (DLPFC) during WM processing in schizophrenia [33,34]. Case-control studies have shown that schizophrenia is associated with higher DLPFC activity during low-demanding WM tasks and lower DLPFC activity during high-demanding WM tasks [35,36], which has also been related to increased schizophrenia PRS [34,37]. Although EM displays the largest effect size among cognitive deficits in schizophrenia [38,39,40,41], fMRI studies on schizophrenia PRS in relation to EM performance are rare. Studies on healthy individuals have shown hypoactivation of temporal, prefrontal, and parietal regions during various EM tasks to be associated with high schizophrenia PRS [42]. Prefrontal hypoactivation along with hypo- and hyperactivation in temporal regions, notably the hippocampus, have been reported in schizophrenia patients compared to healthy controls [3,7]. Although sex differences have been reported in relation to memory in schizophrenia patients, with most studies reporting greater deficits in male patients [30], it is not known whether these differences also relate to underlying brain activation. One fMRI study showed that sex differences in cerebral activations during mental rotation in schizophrenia patients deviated from sex differences observed in healthy volunteers [43], but whether sex differences in brain activation in schizophrenia are related to underlying disease genetics in schizophrenia or in healthy unaffected individuals is not known.

In this study, we investigated the relationship between schizophrenia PRS and brain activity during both WM and EM processing in 351 healthy individuals (167 males and 184 females) within a broad age range, with a four-year follow-up scanning session in a subset of 216 participants (113 males and 103 females). Based on the previous observations of male-specific effects of schizophrenia PRS on cognitive performance in a sample partly overlapping the present one [16], we investigated whether the effect of schizophrenia PRS on brain functioning is also sex-specific, with the hypothesis that the effect of schizophrenia PRS would be male-specific also for brain activity during memory processing.

## 2. Materials and Methods

### 2.1. Participants

Data in the present study come from the brain imaging sample of the longitudinal population-based Betula Prospective Cohort Study on memory, health, and aging conducted in Umeå, Sweden [44] and included data from two cognitive tasks, WM and EM, with scanning at two timepoints for each task. The research was approved by the regional ethical review board at Umeå University (EPN), and all participants gave written informed consent. From the WM task, 344 individuals (163 males and 181 females) aged 25–95 years were included in the present study. Of these 344 individuals, 212 individuals returned for a second fMRI scan four years later. From the EM task, 351 individuals (167 males and 184 females) aged 25–85 years were included in the present study, and 216 of them returned for a second fMRI scan four years later. All participants had European ancestry. Inclusion criteria for all scanning sessions were no contraindications to MRI or notable artifacts in the MRI acquisition, no dementia, and known neurologic or psychiatric disease.

### 2.2. Working Memory Task

The WM task that the participants performed during fMRI acquisition included three conditions: maintenance, manipulation, and control. In all three conditions, lowercase target letters and capital probe letters were presented. The task was divided into six blocks of each condition; each block lasted 27 s and included three trials. The maintenance condition included four target letters that were shown for 2 s followed by a fixation star for 3.5 s. Then, a probe letter was shown for 2.5 s and participants were asked to indicate whether the probe letter was one of the four target letters shown before. The manipulation condition had the same timing and design as the maintenance condition, but it required both maintenance and manipulation of the to-be-remembered information. Two target letters were shown and participants had to indicate whether the probe letter was the subsequent letter in the alphabet to any of the two target letters. The control condition was comparable to the maintenance condition, but participants needed to maintain only one letter in WM, because four identical target letters were shown [45,46].

### 2.3. Episodic Memory Task

The EM task performed during fMRI acquisition was a face-name paired-associates task including 20 blocks of encoding, retrieval, and a control task. The task was divided into six encoding blocks, six retrieval blocks, and eight blocks of an active control task where participants were asked to press a button when a fixation star changed to a circle. During each block, four stimuli were presented for 4 s with a randomized interstimulus interval of 1.5, 2.5, 3, or 4.5 s. During the encoding blocks, digital color photographs of unknown faces that were paired with a common first name were presented, and participants were asked to remember the name associated with each face. During the retrieval blocks, the same faces were presented along with three letters, from which participants were instructed to indicate the letter that corresponds to the first letter of the name previously paired with the face, and participants were instructed to guess if they could not remember the face–name pair [47,48,49].

### 2.4. MRI Acquisition

At both baseline and follow-up, the fMRI data were collected on a 3T General Electric (GE) Discovery MR750 scanner with a 32-channel head coil. The functional images were acquired with a gradient-echo EPI sequence according to the following parameters: TR = 2.0 s, TE = 30 milliseconds, flip angle = 80°, 37 slices (3.9 mm thick), 96 × 96 matrix, and FOV = 25 × 25 cm. To allow for saturation of the fMRI signal, ten dummy scans were collected and discarded prior to experimental image acquisition. E-Prime (Psychology Software Tools) was used for stimulus presentation and recording of responses from a MR-compatible response pad. In addition, structural T1-weighted images were acquired with a 3D fast spoiled gradient echo sequence (180 slices with a 1 mm thickness, TR: 8.2 milliseconds, TE: 3.2 milliseconds, flip angle: 12°, FOV: 25 × 25 cm).

### 2.5. fMRI Analyses

The fMRI data were preprocessed and analyzed using SPM12 (Statistical Parametric Mapping, Wellcome Centre for Human Neuroimaging), implemented in MATLAB R2014b (MathWorks, Natick, MA, USA). SPM was run through an in-house program (DataZ). Preprocessing of the fMRI data included head-movement correction by unwarping and realignment to the first image of each volume. The realigned images were then co-registered to structural T1-weighted images, separately for baseline and follow-up for each participant. The structural images were segmented into gray matter, white matter, and cerebrospinal fluid components. The DARTEL [50] toolbox was used to create a template image for each participant of the baseline and follow-up data and also a group-level DARTEL template. The flow-field files that mapped the transformation from native space to DARTEL template and an affine transformation from DARTEL to Montreal Neurologic Institute (MNI) space was used for normalizing the fMRI data to MNI standard space with a 2-mm resolution and smoothed with an 8-mm full width at half maximum Gaussian kernel. For both fMRI tasks, the data were high-pass filtered (200 s), and voxel-wise general linear models were set up for each participant. In these models, block onsets and durations for each condition from the tasks were included as regressor, modeled as a boxcar, and convolved with a canonical hemodynamic response function (HRF). Six realignment parameters from the motion correction preprocessing steps were included as covariates of no interest to remove movement-related artifacts. 

### 2.6. Genotyping and Construction of Polygenic Risk Scores

The DNA extraction for single-nucleotide polymorphism (SNP) genotyping was performed at the Institute of Human Genetics, University of Bonn, Germany. All DNA samples were genotyped using two types of Illumina arrays: Illumina Omni Express and Omni 1S Bead chip kits. Imputation of the raw genotypes was conducted according to the ENIGMA2 protocol of the ENIGMA Consortium to the 1000 genomes reference panel [51] using minimac tools [52]. Post-imputation quality control (QC) was performed based on genotype call rate < 10%, minor allele frequency (MAF) < 1%, SNP missingness < 5%, and imputation info < 0.8. SNPs with ambiguous strand alignment were removed, as were SNPs within the extended major histocompatibility complex (MHC) region (25–34 Mb on chromosome 6 on the hg19 assembly), due to the high linkage disequilibrium (LD) of this region. PRSs for schizophrenia were calculated in PLINK version 1.9 [53] based on the summary statistics from a GWAS of schizophrenia performed on the PGC2 schizophrenia case-control cohort [10] including 35,476 cases and 46,839 controls, after excluding the current sample. First, LD clumping was performed according to parameters used by the PGC2 [10]: discarding SNPs within 500 kb of, and in, *r*^2^ ≥ 0.1 with another more significant SNP, and excluding SNPs with MAF < 10%. The European sample of the 1000 Genomes Project phase 3 [51] was used as LD reference panel for clumping, after removal of SNPs with genotype call rate < 1% and MAF < 1%. PRSs were based on SNPs located on autosomal chromosomes and calculated for each individual by summing the alleles of the clumped SNPs weighted by the natural log of the odds ratio from the PGC2 GWAS results [10]. The PRSs were constructed by including SNPs with a GWAS *p*-value threshold below 0.05, 0.1, 0.5, and 1, respectively. The explained variance in case-control discrimination has been shown to increase with increasing PRS *p*-value threshold, reaching a plateau at *p* < 0.05 [10,54]. However, the *p*-value threshold explaining largest amount of variance in endophenotypes may not be the same as that for case-control discrimination. Moreover, it has been shown that the *p*-value threshold of *p* ≤ 1 has the highest empirical power in traits with high polygenicity [55], and both schizophrenia [9] and cognitive performance [56] show highly polygenic inheritance. Using Nagelkerke’s pseudo-*R*^2^, we have previously shown that a schizophrenia PRS with the *p*-value threshold of *p* ≤ 1 explained the largest amount of variance in cognitive performance [16], and to avoid multiple testing, we used *p* ≤ 1 for the main fMRI analyses including all clumped SNPs (N = 102,088) from the GWAS summary statistics, and used three PRSs with lower *p*-value thresholds as described above as sensitivity analyses. In addition, we calculated a polygenic score on cognitive performance (Cog-PGS) in the same way as the schizophrenia PRS, using summary statistics from a large multicenter GWAS on cognitive performance measured across at least three cognitive domains including 257,841 individuals [56], and we used the Cog-PGS including all clumped SNPs (N = 102,162) from the GWAS summary statistics (*p*-value threshold ≤ 1).

### 2.7. Statistical Analyses

Subject-level contrast images were generated, comparing the experimental conditions of the scanner task separately for baseline and follow-up data. Contrasts of interest from the WM task were set up for maintenance–control, manipulation–control, and manipulation–maintenance. From the EM task, contrasts of interest were set up for encoding–control and retrieval–control. Second-level analyses investigating the effect of the schizophrenia PRS on brain activation were performed in SPM12. First, using data from the baseline scanning session, second-level analyses were performed using the activation map of the corresponding contrast as explicit mask (*p*-value threshold 0.01 uncorrected) and the PRS as the covariate of interest as well as sex, age, age^2^, and the first five genetic principal components for genetic ancestry as covariates of no interest. Activation maps are shown in Figure S1 (WM) and Figure S2 (EM). To examine the robustness of the results, we then examined whether the results from the baseline scanning session replicated on the four-year follow-up scanning session. For brain regions in which activation was significantly associated with schizophrenia PRS during baseline (at a *p*-value threshold of 0.001 uncorrected, and a minimum of 10 voxels), we created spherical regions of interest (ROI) of 10 mm around the peak voxels. Thereafter, second-level analyses of the schizophrenia PRS as covariate of interest as well as sex, age, age^2^, and the first five genetic principal components for genetic ancestry as covariates of no interest were performed on the follow-up data within these ROIs (*p*-value threshold 0.05 uncorrected). The same ROIs were used to test for interaction effects of PRS with sex in ROI analyses including the interaction term (PRS*sex) as the covariate of interest as well as PRS, sex, age, age^2^, and the first five genetic principal components (*p*-value threshold within ROIs: 0.05 uncorrected). For ROIs showing a significant interaction effect between PRS and sex, we performed sex-stratified ROI analyses including the PRS as the covariate of interest as well as age, age^2^, and the first five genetic principal components (*p*-value threshold within ROIs: 0.001 uncorrected). To also test for interaction effects of PRS with sex in other brain regions, we performed whole-brain analyses including the interaction term (PRS*sex) as the covariate of interest as well as PRS, sex, age, age^2^, and the first five genetic principal components as covariates of no interest (*p*-value threshold: 0.05 FWE corrected). Finally, we performed sex-stratified whole-brain analyses (*p*-value threshold: 0.05 FWE corrected) including the PRS as covariates of interest as well as age, age^2^, and the first five genetic principal components. To investigate the relationship between PRS and performance on cognitive tasks during scanning, linear regression analyses including the PRS as well as sex, age, age^2^, and the first five genetic principal components as covariates were performed in R version 3.5.1. Linear regression analyses were also performed to test if the effect of schizophrenia PRS on brain activation is independent of genetics related to cognitive performance. In these regression analyses, the extracted β values from peak voxels significantly associated with schizophrenia PRSs were used as the dependent variable, with the schizophrenia PRSs as covariates of interest and the Cog-PGS as well as age, age^2^, sex, and the first five genetic principal components as covariates of no interest.

## 3. Results

### 3.1. Behavioral Results

Performance on the scanner tasks as well as other descriptives are summarized in Table 1. Genetic risk for schizophrenia was associated with lower performance on the WM and EM scanner-task (numbers of correct responses) as well as longer response time during EM retrieval in males only (Table 2). Including an interaction term (sex*PRS) in the models revealed significant interaction effects on number of correct responses (with higher effects of PRS in males) during EM retrieval (*t*-value = −2.335, df = 350, *p*-value = 0.0201) and WM maintenance (*t*-value = −2.598, df = 343, *p*-value = 0.0098), but not WM manipulation (*t*-value = −1.183, df = 343, *p*-value = 0.2377). Sex interaction effects on reaction time (with higher effects of PRS in males) were seen for WM maintenance (*t*-value = 2.197, df = 343, *p*-value = 0.0287), as well as trend effects for WM maintenance (*t*-value = 1.798, df = 343, *p*-value = 0.0731) and EM retrieval (*t*-value = 1.927, df = 350, *p*-value = 0.0548).

### 3.2. Memory-Related Brain Activation

**Figure 1:**
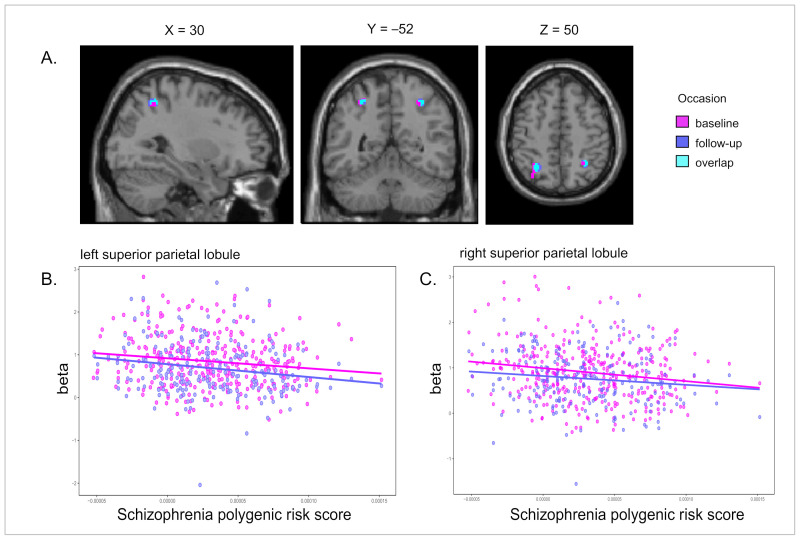
Association between schizophrenia PRS and altered brain activity during episodic memory retrieval at baseline and 4-year follow-up. (**A**) PRS associated with hypoactivation in the bilateral superior parietal lobule during episodic memory retrieval at baseline (voxel threshold: 10, *p*-value threshold: 0.001 uncorrected), with overlapping activation at follow-up (voxel threshold: 10, *p*-value threshold: 0.05 uncorrected in regions-of-interest). (**B**,**C**) Corresponding scatterplot based on peak activity associated with PRS in the left (**B**) and right (**C**) superior parietal lobule.

As expected, schizophrenia’s PRS was most strongly associated with activation in the left DLPFC for the manipulation–maintenance contrast, along with some other brain regions, i.e., less brain activation with a higher PRS (Table 3). None of the correlations between schizophrenia PRS and brain activation during WM at baseline were replicated at follow-up (*p* > 0.05) (Table 3). For the EM task, schizophrenia PRS was significantly associated with activation in the bilateral superior parietal lobule (SPL) during both encoding and retrieval, i.e., less brain activation with higher PRS, and all four clusters replicated at follow-up (Table 3). A few other areas showed an association with schizophrenia PRS, but did not replicate at follow-up (Table 3). Figure 1 shows the results from the retrieval > baseline contrast at baseline (whole-brain analysis) and follow-up (ROI-analysis), with focus on the right SPL cluster. Results from sensitivity analyses using different *p*-value thresholds for PRS can be found in Table S1–3 for both WM and EM, showing weaker effects with lower PRS *p*-value thresholds, which is in line with our previous results of schizophrenia PRS in relation to cognitive performance [16].

To determine whether the effects of schizophrenia genetics are independent of genetic variants with a known effect on cognition, we performed regression analyses, including a polygenic score for cognitive performance (Cog-PGS) as an additional covariate of no interest and the extracted β values from peak values of activation associated with schizophrenia PRS at baseline as the dependent variable. For WM, the effect of schizophrenia PRS on brain activation in the left DLPFC at baseline (manipulation > maintenance) was still significant when including the Cog-PGS (*t*-value = –4.049, df = 343, *p*-value = 0.000064). In addition, the effect of schizophrenia PRS on brain activation in the SPL at baseline was still significant when including the Cog-PGS both during encoding (left SPL: *t*-value = –3.781, df = 350, *p*-value = 0.000184; right SPL: *t*-value = –3.051, df = 350, *p*-value = 0.002461) and retrieval (left SPL: *t*-value = –2.237, df = 350, *p*-value = 0.0259; right SPL: *t*-value = –3.386, df = 350, *p*-value = 0.000793).

### 3.3. Sex-Specific Effects of Schizophrenia Genetics on Functional Brain Activity

**Figure 2:**
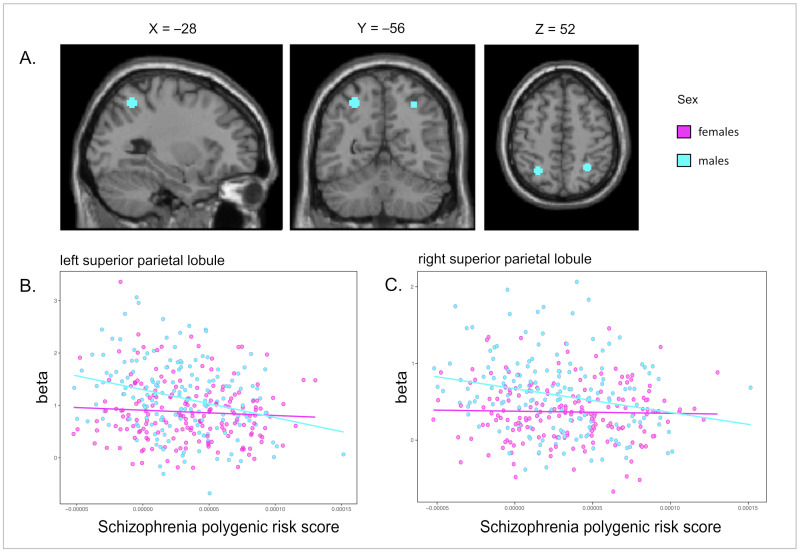
Association between schizophrenia PRS and altered brain activity during episodic memory retrieval in males and females at baseline. (**A**) PRS associated with hypoactivation in the bilateral superior parietal lobule during episodic memory retrieval (voxel threshold: 10, *p*-value threshold 0.001 uncorrected in regions-of-interest). The association was significant in males only (*p* = 0.00007 in left and *p* = 0.00044 in right superior parietal lobule). (**B**,**C**) Corresponding scatterplot based on the peak activity associated with PRS in the left (**B**) and right (**C**) superior parietal lobule.

We first examined sex differences within the brain areas where we found the strongest effect of the schizophrenia PRS: hypoactivation of the left and right SPL during EM and the left DLPFC during WM processing. Adding an interaction term (PRS*sex) to the model revealed that there was a significant interaction with schizophrenia PRS and sex in the left and right SPL both during episodic encoding (left SPL: *t*-value = 2.31, *p*-value = 0.011; right SPL: *t*-value = 2.35, *p*-value = 0.010) and retrieval (left SPL: *t*-value = 2.82, *p*-value = 0.003; right SPL: *t*-value = 1.96, *p*-value = 0.025). For the DLPFC during WM (manipulation–maintenance), a trend in the same direction as for EM was observed (i.e., stronger effect of PRS on hypoactivation in males; *t*-value = 1.39, *p*-value = 0.079). The interaction effects (PRS*sex) in the left SPL remained significant even when the Cog-PGS was included in the models, both during encoding (*t*-value = 2.23, *p*-value = 0.027) and retrieval (*t*-value = 2.64, *p*-value = 0.009), but not in the right SPL (encoding: *t*-value = 1.13, *p*-value = 0.258; retrieval: *t*-value = 1.30, *p*-value = 0.193). Sex-stratified analyses within the SPL ROIs showed a significant negative effect of schizophrenia PRS on brain activation in males, both during encoding (*p*-value left SPL = 0.00005; *p*-value right SPL = 0.00048) and retrieval (*p*-value left SPL = 0.00007; *p*-value right SPL = 0.00044), whereas no effect was seen in females (all *p*-values > 0.01) (Table 4). Figure 2 shows the association between schizophrenia PRS and EM-related brain activity for males and females in the left and right SPL.

As an additional analysis, we examined sex interactions in FWE-corrected whole brain analyses during WM and EM at baseline. During EM retrieval, these analyses confirmed a significant interaction with schizophrenia PRS and sex in the right SPL (*t*-value = 5.02, *p*-value _FWE-corrected_: 0.003) as well as in the right ventral diencephalon (*t*-value = 4.54, *p*-value _FWE-corrected_: 0.033), while no significant effects were seen during EM encoding or WM processing. Sex-stratified whole-brain analyses showed that the PRS was significantly associated with lower brain activation in one brain region during episodic retrieval in males, the right SPL (*t*-value = 4.88, *p*-value _FWE-corrected_: 0.009, number of voxels: 68). The interaction between PRS and sex in the SPL did not reach FWE-corrected significance at follow-up.

We also examined whether schizophrenia PRS was associated with age-related changes in brain activation in any of the identified regions. We found no interaction effects between schizophrenia PRS and age at baseline (*p* > 0.05).

## 4. Discussion

The current work constitutes a comprehensive investigation of the effect of schizophrenia PRS on memory-related brain functioning. We first replicated past reports of a link between schizophrenia genetics and decreased DLPFC activation during WM processing and extended these previous results [33,34,37] to hold across a broader age-range including older participants. Moreover, we identified a robust effect of decreased brain activation in the bilateral SPL during EM encoding and retrieval. The SPL effect was male-specific, and a similar trend was seen for the DLPFC, which is in line with our recent findings of a male-specific effect of schizophrenia PRS on lifespan cognitive functioning in healthy individuals [16]. To our knowledge, the results shown here are the first to demonstrate a sex-specific effect of schizophrenia genetics on brain activation.

WM is dependent on DLPFC functionality [57], which is known to be hypoactive in schizophrenia patients [34,36,37]. We observed that schizophrenia PRS was associated with hypoactivation in the left DLPFC during manipulation, but not during the less demanding maintenance condition. This results pattern is in line with the DLPFC inefficiency hypothesis, stating that individuals with cognitive impairment are not able to recruit the DLPFC to a sufficient degree during high-demanding tasks [45]. While Miller et al., found a bilateral hypoactivation of the DLPFC in relation to schizophrenia PRS [33], the current study and some others report hypoactivity only in the left DLPFC to be significantly associated with schizophrenia PRS [34,37], and case-control fMRI studies have also shown that altered brain activation in schizophrenia was stronger in the left DLPFC [35,36,58,59]. Nyberg et al., reported that the left DLPFC was most sensitive to elevated WM demands in individuals showing cognitive constraints [45], which may explain the fact that schizophrenia genetics has the largest effect on the left DLPFC. Notably, there was a trend toward an interaction effect between sex and PRS in the left DLPFC (*p* = 0.079), indicating that the effect of PRS on hypoactivation in the left DLPFC during WM retrieval may be stronger in males. The lack of replication at follow-up may be due to the effect being too weak to be detected in the smaller sample at follow-up, where power was reduced due to an almost 50% drop in sample size. In sum, the present WM results confirm previous suggestions of DLPFC inefficiency during WM processing as a genetically modulated intermediate phenotype of schizophrenia.

Schizophrenia PRS was linked to hypoactivation in the bilateral SPL during EM encoding and retrieval, with similar patterns at follow-up, supporting the robustness of this relation. Hypoactivation of the SPL in schizophrenia patients is not among the most common case/control findings during EM, where a primary focus has been the hippocampus (with inconsistent results) [3]. However, SPL hypoactivation in schizophrenia patients relative to controls has been reported in some previous EM fMRI studies [60,61], but sex differences were not addressed in these studies. In one fMRI study including a sex by diagnosis interaction, Jimenez et al. [43] found that sex differences in brain activation during a mental rotation fMRI task were not the same for schizophrenia patients as for healthy controls. They found significant activations in the inferior and superior parietal cortex in healthy men and women with schizophrenia that were not seen in men with schizophrenia or in healthy women [43]. Increased activity in the posterior parietal cortex, including the inferior and superior parietal cortex, is one of the most robust findings in fMRI studies investigating the neural correlates of EM encoding [62] and retrieval [63,64,65]. Corbetta and Shulman [66] suggested that the SPL supports goal-directed (top-down) modulation of attention, which is reinforced by the Attention to Memory Model (AToM) by Cabeza et al. [67], stating that the SPL is associated with active memory-retrieval search (top-down attention). Further, deficits in top-down attentional modulation have previously been reported in schizophrenia patients [68,69,70]. Thus, our finding of hypoactivity in the bilateral SPL in relation to schizophrenia PRS in males may represent a genetically modified deficit in top-down attentional modulation, that may in turn translate into impaired self-initiated semantic encoding and retrieval strategies, leading to worse memory performance of male schizophrenia patients [60]. To what extent a more general dysfunction of parietal attention networks also impacts cognitive domains other than episodic memory remains to be examined. In support of a more general dysfunction, our previous study showed that the male-specific effect of schizophrenia PRS was generalized across all three cognitive tests included in that study: visuospatial ability, semantic memory, and EM, as well as on school grades across six subjects [16].

Taken together, our results highlight the importance of considering sex differences when studying the impact of disease genetics on intermediate phenotypes, and open up for further examination the interactions between schizophrenia risk genes and sex hormones or other sex-related biological processes. One limitation of our study is the lack of an independent replication sample. However, based on a previous study of this dataset showing that the activation patterns at baseline had high reproducibility at the follow-up session [49], we used the follow-up dataset as a within-sample replication to see if the effect of schizophrenia PRS at baseline shows similar patterns in the follow-up dataset, and for the SPL findings we could replicate the same trend as seen at baseline. This approached justifies the analyses at an uncorrected level (*p* < 0.001, cluster level threshold > 10 voxels). It should also be noted that the effect of schizophrenia PRS*sex on SPL activation also survived multiple comparisons in the FWE-corrected whole-brain analyses. Still, future efforts seeking to replicate our sex-specific effect of schizophrenia genetics on brain phenotypes in independent samples of healthy individuals as well as schizophrenia patients are warranted.

## 5. Conclusions

In summary, we were able to replicate the previously reported link between schizophrenia genetics and hypoactivation in the DLPFC during WM processing, and found a robust effect in the SPL (i.e., less brain activation with higher PRS), during both encoding and retrieval of EM, that was male-specific. These results suggest superior parietal regions as a novel male-specific genetic intermediate phenotype in schizophrenia, potentially reflecting an impact on top-down modulation of attention that contributes to the more severe memory dysfunctions seen in male schizophrenia patients. The current results of sex differences in the impact of schizophrenia genetics on cognition-related brain functioning open up this topic for further examinations that may ultimately facilitate the development of sex-specific pharmacological treatment options.

## Figures and Tables

**Table 1:** Descriptive statistics for baseline and follow-up.

	Baseline	Follow-Up	t/χ^2^ Value	*p*-Value
**Working Memory**				
Age, Mean (SD)	63.13 (13.46)	64.94 (13.01)	−1.57	0.12 ^a^
Sex, N females (males)	181 (163)	101 (111)	1.11	0.29 ^b^
RT, Maintenance	1.35 (0.27)	1.30 (0.26)	2.26	0.02 ^a^
RT, Manipulation	1.45 (0.32)	1.40 (0.32)	1.57	0.12 ^a^
Hits - FA, Maintenance	7.93 (1.58)	8.15 (1.33)	−1.73	0.08 ^a^
Hits - FA, Manipulation	6.42 (2.50)	6.47 (2.36)	−0.25	0.80 ^a^
**Episodic Memory**				
Age, Mean (SD)	63.10 (13.25)	65.01 (12.97)	−1.69	0.09 ^a^
Sex, N females (males)	184 (167)	103 (113)	1.02	0.31 ^b^
RT, Retrieval, Mean (SD)	2.62 (0.34)	2.65 (0.44)	−0.89	0.37^a^
Hits, Retrieval, Mean (SD)	14.29 (4.42)	16.10 (4.00)	−5.00	8.02 × 10^−7 a^

RT = mean response time (in seconds), SD = standard deviation, ^a^: Welch Two Sample *t*-test (baseline vs. follow-up), ^b^: Chi-square test (baseline vs. follow-up).

**Table 2:** Relationship between scanner task performance and polygenic risk for schizophrenia at baseline.

		Adjusted *R*^2^	SE	*t*-Value (df)	*p*-Value
Working Memory					
RT, Maintenance	All	−0.0022	0.0135	0.248 (343)	0.804
	Males	0.0070	0.0222	1.524 (162)	0.129
	Females	0.0019	0.0165	−1.215 (180)	0.226
RT, Manipulation	All	−0.0026	0.0176	0.111 (343)	0.912
	Males	0.0023	0.0284	1.174 (162)	0.242
	Females	−0.0009	0.0220	−0.897 (180)	0.371
Hits - FA, Maintenance	All	0.0438	0.0866	−1.088 (343)	0.277
	Males	0.0257	0.1361	−2.357 (162)	0.020 *
	Females	−0.0013	0.1116	0.866 (180)	0.388
Hits - FA, Manipulation	All	0.1964	0.1209	−2.537 (343)	0.012 *
	Males	0.0240	0.1746	−2.489 (162)	0.014*
	Females	0.0020	0.1688	−1.219 (180)	0.224
**Episodic Memory**					
RT, Retrieval	All	0.0031	0.0172	1.531 (350)	0.127
	Males	0.0186	0.0256	2.203 (166)	0.029 *
	Females	−0.0040	0.0228	0.190 (183)	0.849
Hits, Retrieval	All	0.0034	0.2097	−1.660 (350)	0.098
	Males	0.0319	0.3064	−2.904 (166)	0.004 *
	Females	−0.0034	0.2830	0.045 (183)	0.964

RT = response time, FA = false alarm (falsely identified letters). SE = standard error. df = degrees of freedom (N–1). Linear regression analyses included sex (when applicable), age, age^2^, and the first 5 genetic principal components for genetic ancestry as covariates of no interest. * = *p* < 0.05.

**Table 3:** Peak values of activation associated with polygenic risk for schizophrenia during working memory and episodic memory (*p*-value threshold: 0.001, voxel threshold: 10) from whole-brain analyses at baseline and ROI analyses at follow-up (*p*-value threshold: 0.05). N = number of voxels.

	Coordinates	Brain Region	*t*-Value (df)	*p*-Value	N	Follow-Up, Coordinates, *p* (N)
Working Memory						
Manip-Ctrl						
Neg cor PRS	4 −96 4	No area	3.87 (343)	0.000064	31	ns
	−6 −6 14	L Thalamus	3.64 (343)	0.000156	41	ns
	6 −62 −36	CVL	3.55 (343)	0.000219	45	ns
	40 −86 −2	R IOG	3.50 (343)	0.000268	17	ns
	−46 −44 22	L cerebral WM	3.45 (343)	0.000315	17	ns
Manip-Maint						
Neg cor PRS	−44 40 30	L DLPFC	4.20 (343)	0.000017	80	ns
	−52 22 −6	No area	3.66 (343)	0.000145	60	ns
	−36 −76 40	L angular gyrus	3.23 (343)	0.000669	14	ns
	−2 −26 −8	No area	3.21 (343)	0.000737	10	ns
**Episodic memory**						
Enc-Ctrl						
Neg cor PRS	−30 −52 56	L SPL	4.04 (350)	0.000034	137	−28 −54 52, 0.004600 (44)
	34 −78 32	No area	3.36 (350)	0.000430	19	ns
	30 −48 52	R SPL	3.35 (350)	0.000454	28	34 −46 52, 0.010790 (26)
Retr-Ctrl						
Neg cor PRS	54 −72 6	R IOG	3.80 (350)	0.000085	47	ns
	−28 −56 52	L SPL	3.62 (350)	0.000171	95	−26 −54 56, 0.004511 (80)
	0 −96 16	No area	3.59 (350)	0.000191	59	ns
	30 −52 50	R SPL	3.50 (350)	0.000268	32	34 −50 52, 0.013565 (45)
	20 −78 52	No area	3.35 (350)	0.000449	17	ns
	−20 −82 42	No area	3.29 (350)	0.000551	30	ns
Pos cor PRS	34 −12 −28	R HC	3.41 (350)	0.000361	14	ns

Manip-Ctrl = Manipulation-Control, Manip-Maint = Manipulation-Maintenance, Enc-Ctrl = encoding-control, Retr-Ctrl = retrieval-control. Cor = correlation with schizophrenia PRS, CVL = cerebellar vermal lobules, IOG = inferior occipital gyrus, WM = white matter, DLPFC = dorsolateral prefrontal cortex, SPL = superior parietal lobule, HC = hippocampus. Neg = negative. Pos = positive. df = degrees of freedom (N–1).

**Table 4:** Peak values of activation negatively associated with polygenic risk for schizophrenia during episodic memory from sex-stratified analyses within the SPL regions of interest.

	Sex	Coordinates	Brain Region	*t*-Value (df)	*p*-Value	N Voxels with *p* < 0.001
Enc-Ctrl	Males	−30 −54 56	L SPL	3.99 (166)	0.00005 *	81 of 81
		34 −50 54	R SPL	3.36 (166)	0.00048 *	75 of 81
	Females	−26 −54 56	L SPL	2.09 (183)	0.01893	0 of 81
		30 −52 50	R SPL	2.08 (183)	0.01927	0 of 81
Retr-Ctrl	Males	−28 −56 52	L SPL	3.92 (166)	0.00007 *	81 of 81
		32 −50 54	R SPL	3.39 (166)	0.00044 *	81 of 81
	Females	−30 −60 50	L SPL	1.53 (183)	0.06169	0 of 81
		30 −54 50	R SPL	1.96 (183)	0.02547	0 of 81

Enc-Ctrl = encoding-control, Retr-Ctrl = retrieval-control, R = right, L = left, SPL = superior parietal lobule. df = degrees of freedom (N–1). * = *p* < 0.001.

## Data Availability

Not applicable.

## Supplementary Materials

The following supporting information can be downloaded at: https://www.mdpi.com/article/10.3390/genes13030412/s1, Figure S1: Activation masks from the working memory task at baseline; Figure S2: Activation masks from the episodic memory task at baseline; Table S1: Peak values of activation associated with schizophrenia PRS (*p* < 0.5) during working memory and episodic memory; Table S2: Peak values of activation associated with schizophrenia PRS (*p* < 0.1) during working memory and episodic memory; Table S3: Peak values of activation associated with schizophrenia PRS (*p* < 0.05) during working memory and episodic memory.
