# Supplementary material for: Polygenic Risk for Schizophrenia Has Sex-Specific Effects on Brain Activity during Memory Processing in Healthy Individuals
Source: Genes (Basel). 2022 Feb 24;13(3):412. doi: 10.3390/genes13030412 (PMC8950000; doi:10.3390/genes13030412)
Supplement: Supplementary file 1 [file genes-13-00412-s001.zip › genes-1576170-supplementary.pdf]

Supplementary Materials

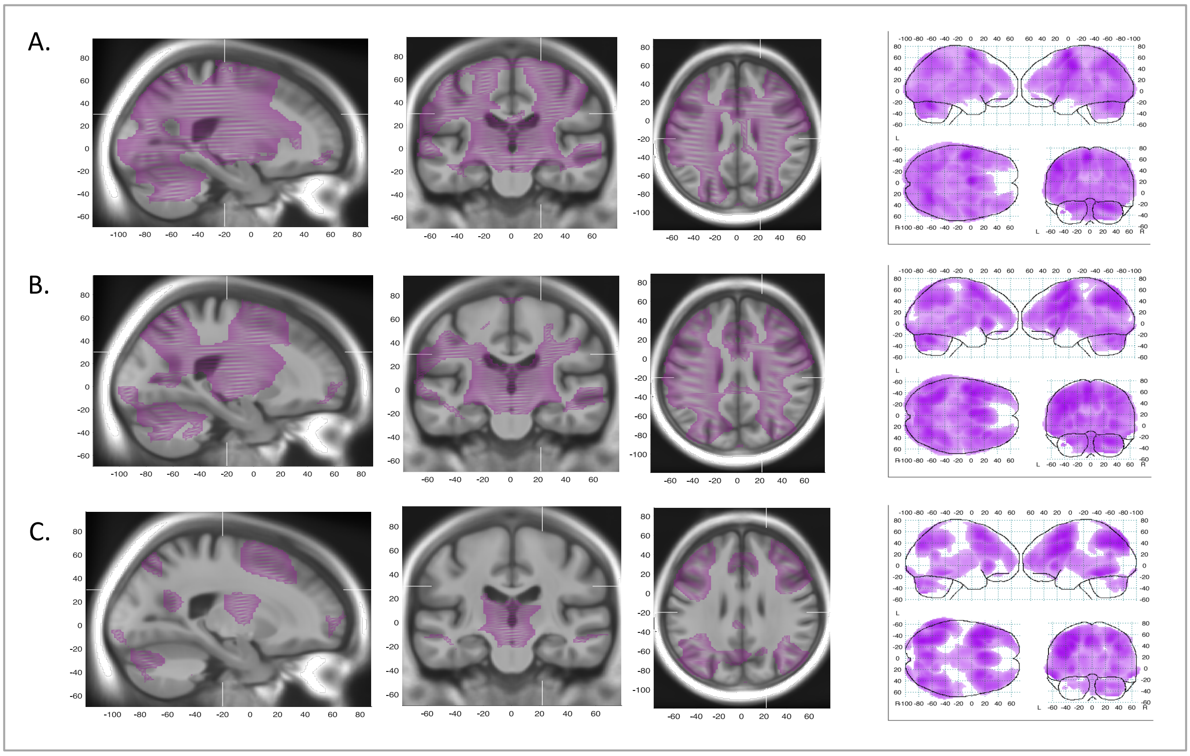

Figure S1. Activation masks from the working memory task at baseline (*p*-value threshold 0.01). Activation refers to the average activation of the cohort (N = 344). **A.** Maintenance > Control. **B.** Manipulation > Control. **C.** Manipulation > Maintenance.

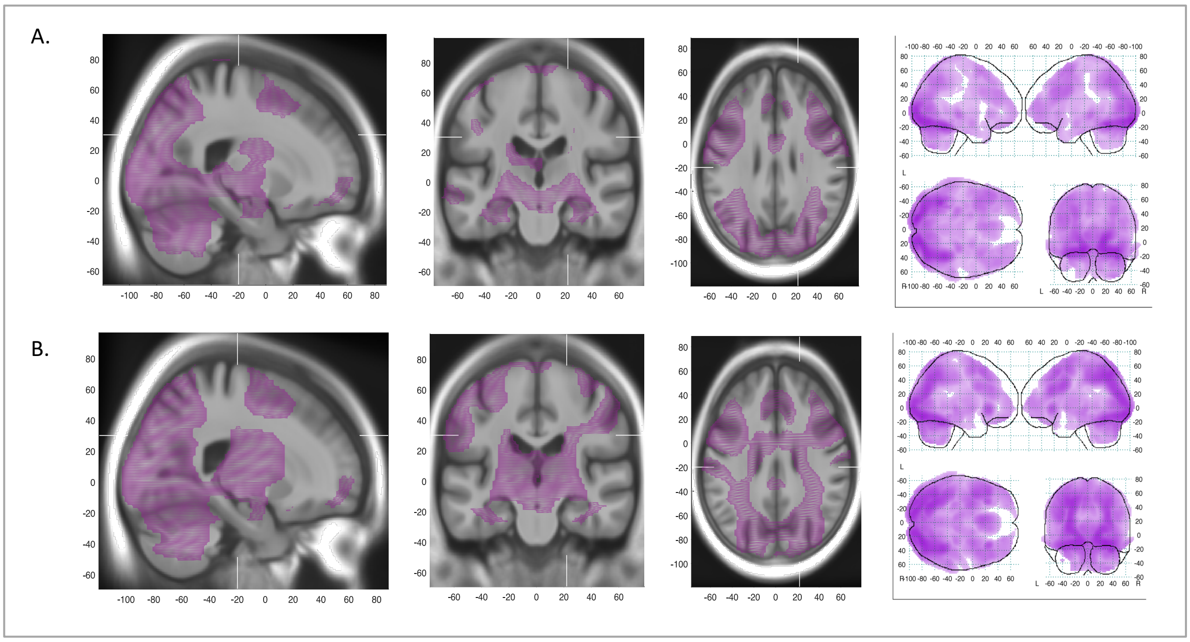

Figure S2. Activation masks from the episodic memory task at baseline (*p*-value threshold 0.01). Activation refers to the average activation of the cohort (N = 351). **A.** Encoding > Control. **B.** Retrieval > Control.

Table S1. Peak values of activation associated with schizophrenia PRS (***p* < 0.5**) during working memory and episodic memory (*p*-value threshold: 0.001, voxel threshold: 10) from whole-brain analyses at baseline and ROI analyses at follow-up (*p*-value threshold: 0.05, voxel threshold 10). N = number of voxels.

	**Coordinates**	**Brain region**	***t*-value (df)**	***p*-value**	**N**	**Replication Follow-up, coordinates, *p* (N)**
**Working memory**						
Manip-Ctrl						
Neg cor PRS	4 -96 4	No area	4.01 (343)	0.000038	36	ns
	6 -62 -36	CVL	3.54 (343)	0.000225	41	ns
	40 -86 -2	R IOG	3.45 (343)	0.000280	16	ns
	-46 -44 22	L cerebral WM	3.37 (343)	0.000416	10	ns
	-6 -6 14	L Thalamus	3.36 (343)	0.000436	11	ns
Manip-Maint						
Neg cor PRS	-44 40 30	L DLPFC	4.17 (343)	0.000020	79	ns
	-52 22 -6	No Area	3.63 (343)	0.000165	57	ns
	-34 -76 42	L angular gyrus	3.31 (343)	0.000517	27	ns
**Episodic memory**						
Enc-Ctrl						
Neg cor PRS	-28 -52 56	L SPL	4.01 (350)	0.000037	134	-26 -54 52, 0.004446 (51)
	-54 -52 24	L cerebral WM	3.37 (350)	0.000414	21	ns
	30 -48 52	R SPL	3.37 (350)	0.000420	35	34 -46 52, 0.014096 (21)
	34 -78 32	No area	3.35 (350)	0.000454	19	ns
	38 -62 -30	R Cerebellum	3.25 (350)	0.000624	12	ns
Retr-Ctrl						
Neg cor PRS	54 -72 6	R IOG	3.75 (350)	0.000102	39	ns
	30 -52 50	R SPL	3.57 (350)	0.000207	43	34 -50 52, 0.011660 (45)
	-28 -56 52	L SPL	3.52 (350)	0.000242	76	-26 -54 56, 0.003627 (81)
	0 -96 16	No Area	3.52 (350)	0.000247	39	ns
	-20 -82 42	No area	3.23 (350)	0.000669	17	ns
Pos cor PRS	36 -10 -28	R HC	3.44 (350)	0.000326	16	ns

Cor PRS = correlation with schizophrenia PRS, Manip-Ctrl = Manipulation-Control, Manip-Maint = Manipulation-Maintenance, Enc-Ctrl = encoding-control, Retr-Ctrl = retrieval-control, CVL = cerebellar vermal lobules, IOG = inferior occipital gyrus, WM = white matter, DLPFC = dorsolateral prefrontal cortex, SPL = superior parietal lobule, HC = hippocampus. Neg = negative. Pos = positive. df = degrees of freedom (N – 1).

Table S2. Peak values of activation associated with schizophrenia PRS (***p* < 0.1**) during working memory and episodic memory (*p*-value threshold: 0.001, voxel threshold: 10) from whole-brain analyses at baseline and ROI analyses at follow-up (*p*-value threshold: 0.05, voxel threshold 10). N = number of voxels.

	Coordinates	Brain region	*t*-value (df)	p-value	N	Replication Follow-up, *p*
**Working memory**						
Manip-Ctrl						
Neg cor PRS	4 -96 4	No area	4.55 (343)	0.000004	125	ns
	6 -62 -36	CVL	3.91 (343)	0.000056	128	ns
	-42 52 18	No area	3.60 (343)	0.000186	29	ns
	40 -86 -2	R IOG	3.55 (343)	0.000222	22	ns
	-8 -6 14	L Thalamus	3.45 (343)	0.000317	21	ns
	-44 -42 22	L cerebral WM	3.40 (343)	0.000374	19	ns
	-10 -60 -37	L Cerebellum	3.29 (343)	0.000506	11	ns
	-42 8 58	No area	3.26 (343)	0.000605	15	ns
Manip-Maint						
Neg cor PRS	-44 40 30	L DLPFC	4.13 (343)	0.000023	53	ns
**Episodic memory**						
Enc-Ctrl						
Neg cor PRS	-30 -52 56	L SPL	3.72 (350)	0.000118	107	ns
	-32 -76 -28	L Cerebellum	3.66 (350)	0.000146	59	ns
	22 -78 50	R SPL	3.40 (350)	0.000376	20	ns
	-28 -86 26	L MOG	3.27 (350)	0.000594	12	ns
	34 -80 28	R MOG	3.17 (350)	0.000827	13	ns
Retr-Ctrl						
Neg cor PRS	20 -76 52	R SPL	3.76 (350)	0.000099	51	ns
	2 -96 6	No area	3.55 (350)	0.000219	58	ns
	-34 -66 52	L angular gyrus	3.45 (350)	0.000319	63	ns
	54 -74 6	R IOG	3.37 (350)	0.000414	14	ns
	30 -52 50	No area	3.36 (350)	0.000430	16	ns
Pos cor PRS	32 -14 -28	R HC	3.44 (350)	0.000327	15	ns

Cor PRS = correlation with schizophrenia PRS, Manip-Ctrl = Manipulation-Control, Manip-Maint = Manipulation-Maintenance, Enc-Ctrl = encoding-control, Retr-Ctrl = retrieval-control, CVL = cerebellar vermal lobules, IOG = inferior occipital gyrus, WM = white matter, DLPFC = dorsolateral prefrontal cortex, SPL = superior parietal lobule, MOG = middle occipital gyrus, HC = hippocampus. Neg = negative. Pos = positive. df = degrees of freedom (N – 1).

Table S3. Peak values of activation associated with schizophrenia PRS (***p* < 0.05**) during working memory and episodic memory (*p*-value threshold: 0.001, voxel threshold: 10) from whole-brain analyses at baseline and ROI analyses at follow-up (*p*-value threshold: 0.05, voxel threshold 10). N = number of voxels.

	Coordinates	Brain region	*t*-value (df)	*p*-value	N	Replication Follow-up, *p*
**Working memory**						
Maint-Ctrl						
Pos cor PRS	20 12 70	No area	4.30 (343)	0.000011	22	ns
Manip-Ctrl						
Neg cor PRS	-18 -100 -4	L occipital pole	3.74 (343)	0.000108	38	ns
	-34 8 64	No area	3.47 (343)	0.000289	10	ns
Manip-Maint						
Neg cor PRS	-20 -100 -6	L occipital pole	3.88 (343)	0.000063	16	ns
	38 -78 -26	R cerebellum exterior	3.69 (343)	0.000130	102	ns
	-6 -24 -16	L ventral DC	3.59 (343)	0.000190	51	ns
	-44 42 28	L middle frontal gyrus	3.51 (343)	0.000253	20	ns
	0 -36 -18	4th ventricle	3.43 (343)	0.000345	10	ns
**Episodic memory**						
Retr-Ctrl						
Pos cor PRS	-18 -98 -6	L occipital pole	3.37 (350)	0.000424	10	ns

Cor PRS = correlation with schizophrenia PRS, Maint-Ctrl = Maintenance-Control, Manip-Ctrl = Manipulation-Control, Manip-Maint = Manipulation-Maintenance, Retr-Ctrl = retrieval-control, Neg = negative. Pos = positive. df = degrees of freedom (N – 1).
